# The Role of the Pulmonary Artery Obstruction Index Ratio in Predicting the Clinical Course of Pulmonary Embolism

**DOI:** 10.3390/jcm14051673

**Published:** 2025-03-01

**Authors:** Serap Atik, Recai Ergün, Dilek Ergün, Ecem Narin Çopur, Abidin Kılınçer, Muslu Kazım Körez

**Affiliations:** 1 Department of Pulmonary Medicine, Iğdır Dr. Nevruz Erez State Hospital, Iğdır 76000, Turkey; 2 Department of Pulmonary Medicine, Faculty of Medicine, Selcuk University, Konya 42130, Turkey; dilek.ergun@selcuk.edu.tr; 3 Department of Pulmonary Medicine, Dr. Yaşar Eryılmaz Doğubeyazıt State Hospital, Ağrı 04402, Turkey; dr.ecemnarin@gmail.com; 4 Department of Radiology, Faculty of Medicine, Selcuk University, Konya 42130, Turkey; abidin.kilincer@selcuk.edu.tr; 5 Department of Biostatistics, Faculty of Medicine, Selcuk University, Konya 42130, Turkey; kkorez@selcuk.edu.tr

**Keywords:** acute pulmonary embolism (APE), sPESI, CTOI, mortality, IVSD, PAD/AoD, IVCR

## Abstract

**Background/Objective:** This study aimed to investigate the relationship between the pulmonary arterial computed tomography obstruction index ratio (CTOI) and the simplified pulmonary embolism severity index (sPESI), one of the clinical probability scoring modalities, in determining the severity of PE and to determine whether CTOI is a mortality marker. **Methods:** The study included 117 patients diagnosed with PE via computed tomography pulmonary angiography (CTPA). The CTOI was determined according to the localization of the embolus and the obstruction caused by the embolus in the vessel. Patients were divided into two groups, namely low-risk and high-risk groups, according to their sPESI values. Patient deaths up to six months after PE diagnosis were recorded. **Results:** According to the sPESI classification, although the CTOI was higher in the high-risk group compared to the low-risk group, no significant difference was found between the groups. The mortality rate was significantly higher in the high-risk group. After six months of follow-up, there was no difference in the CTOI rate between the patients who died and those who survived. **Conclusions:** Although CTPA is the gold standard for diagnosing PE, it would be more appropriate to use it together with clinical findings to determine the severity of the disease. Further evaluation is needed to investigate the usefulness of the obstruction index and CT findings of right ventricular dysfunction for classifying patient risk and determining therapeutic options.

## 1. Introduction

Pulmonary embolism (PE) patients may have different outcomes and require different intensities of clinical care. It is therefore necessary to perform risk assessments to decide on the appropriate management strategy [1]. This risk assessment can be based on clinical, biochemical, and imaging parameters [2]. The simplified pulmonary embolism severity index (sPESI), a clinical probability scoring system using clinical parameters to determine prognosis in PE patients, is important in determining the clinical course [3]. Right ventricular (RV) failure due to a massive embolism is a critical determinant of the clinical severity and outcome of PE [4]. Computed tomography pulmonary angiography (CTPA) and transthoracic echocardiography (TTE) can determine the degree of RV failure. An increased main pulmonary artery diameter (PAD), pulmonary artery to aortic diameter ratio (PAD/AoD), interventricular septal deviation (IVSD), and inferior vena cava reflux (IVCR) are among the parameters that can be evaluated using CTPA [5]. Qanadli et al. created the pulmonary arterial computed tomography obstruction index ratio (CTOI) in order to quantify the degree of pulmonary arterial obstruction and to determine whether it correlated with pulmonary artery pressure and right ventricle function [6].

The primary aim of our study was to evaluate the relationship between CTOI and sPESI and to determine whether it is a predictor of mortality. The secondary aim was to analyze whether there was a significant association between CTOI and signs of right ventricular dysfunction (IVSD, PAD/AoD ratio, and IVCR). By integrating these assessments, this approach aims to enhance risk stratification, guide clinical decision making, and optimize patient outcomes through timely and targeted interventions.

## 2. Materials and Methods

### 2.1. Study Populations

The data of 1035 patients who were admitted to Selçuk University Faculty of Medicine Chest Diseases Department with a provisional diagnosis of APE between 2019 and 2022 were retrospectively obtained from the hospital digital record system. Patients with insufficient contrast transmission on CTPA, suspicious filling defects, embolism diagnosed with ventilation–perfusion (V/Q) scintigraphy, and the presence of chronic thromboembolic pulmonary hypertension; patients who were pregnant and under the age of 18; and patients who had undergone lung surgery or had incomplete clinical/laboratory findings were excluded. The study included 117 patients who were diagnosed with acute pulmonary embolism (APE) via computed tomography pulmonary angiography (CTPA) and had been treated as inpatients.

The patients’ symptoms, physical examination findings, CTPA, Doppler ultrasound, and laboratory results were recorded on an evaluation form using hospital data processing records. Patients were classified according to the simplified Pulmonary Embolism Severity Index (sPESI). Those with an sPESI score of 0 were considered low risk, while those with an sPESI score of 1 or higher were classified as high risk.

Patients diagnosed with acute pulmonary embolism (APE) were treated according to current clinical guidelines, including anticoagulant therapy (Vitamin K antagonists, new oral anticoagulants), thrombolytic therapy (tissue plasminogen activator), and supportive treatments as needed (e.g., oxygen therapy, vasopressor therapy, mechanical ventilation). Patients with severe clinical conditions were monitored in the intensive care unit (ICU), while stable patients were managed in the ward. After discharge, all patients were scheduled for follow-up in the outpatient clinic, and mortality data were recorded via the Ministry of Health’s Death Notification System. Due to the retrospective design of the study, informed consent was waived.

### 2.2. Radiological Evaluation

All patients also underwent standard CTPA within 6 h. A total of 60 mL of intravenous iohexol was administered at a 4 mL/s flow rate. CTPA was performed using the bolus tracking technique by putting the region of interest to the pulmonary artery with a 256-MDCT (Multidetector Computed Tomography) scanner (Siemens Somatom Definition Flash, Erlangen, Germany). The scan parameters were as follows: 120 kV, a 1 mm slice thickness, and a 1.5 pitch value. Coronal reformatted images and images with an axial/coronal 20 mm thick maximum intensity projection were also created. The images were evaluated in the mediastinal window by a radiologist specialized in chest radiology. The CTOI was calculated according to the localization of the embolus on Computed Tomography Pulmonary Angiography and the occlusion in the vessel [6]. IVSD, IVCR, and PAD/AoD were examined to determine right ventricular dysfunction.

The pulmonary arterial computed tomography obstruction index ratio (CTOI) score is calculated by evaluating the degree of obstruction in each pulmonary artery segment using CT angiography. Each lung consists of 10 segments, making a total of 20 segments. Each segment is graded based on the degree of obstruction: 0 (no clot), 1 (partial obstruction), or 2 (complete obstruction). After summing the scores of the segments, the total score is obtained, with a maximum score of 40. This score is used to evaluate the clot burden and severity of the disease [6].

The interventricular septal deviation was subjectively assessed by the radiologist conducting the examination. The septum was considered to have septal deviation if it appeared flattened or convexly bowed to protect the left ventricle in any image [7].

The diameters of the main pulmonary artery and the ascending aorta were measured at the level where the right pulmonary artery continues with the main pulmonary artery and sweeps the midline. Subsequently, the ratio of the diameter of the pulmonary artery to the diameter of the ascending aorta was calculated [8].

The contrast reflux was recorded when contrast material was detected in the IVC and hepatic vein [9].

To evaluate the relationship between sPESI and radiological parameters, radiological findings (CTOI, AoD, PAD, PDA/AoD ratio, IVSD, and IVCR) were compared between low- and high-risk groups. Additionally, the presence of other radiological markers associated with mortality was also analyzed.

### 2.3. Statistical Analyses

All the statistical analyses were performed in R version 4.1.2 (www.r-project.org, accessed on 28 January 2025) with the help of a statistical programming language. Before the analyses, the normality of the data was checked using Shapiro–Wilk’s normality test, Q-Q graphs, and Levene’s test. Numerical variables are presented as means ± standard deviations or medians (quartiles), and categorical variables are presented as frequencies (n) and percentages (%). Statistical differences in the patients’ demographic, clinical, and radiologic findings and blood parameters, according to their sPESI groups and mortality status, were analyzed using the independent sample *t*-test, Welch’s test, or the Mann–Whitney U test for numerical variables and using Yates continuity-corrected chi-square and Fisher’s exact test for categorical variables. In addition, the CTOI values of clinical and radiologic findings were compared using the Mann–Whitney U test. The effect sizes were calculated using Cohen’s *d* for the independent samples *t*-test and Welch’s *t*-test, the Phi (*ϕ*) coefficient for the Chi-square test, and the Rank biserial correlation coefficient (*r*_b_) for the Mann–Whitney U test. The level of significance was set at 5%.

## 3. Results

The demographic data of the patients are summarized in Table 1. The study included 117 patients (50 males, 67 females) with a mean age of 63.29 ± 16.29 (24–115) years. There were 28 patients in the low-risk group (sPESI = 0) and 89 patients in the high-risk group (sPESI ≥ 1). The gender distribution was equal between groups and there was no difference in BMI. The mean age (66.71 ± 15.83 vs. 52.43 ± 12.79, *p* = 0.001, Cohen’s *d* = 0.94) and presence of comorbidities were higher in high-risk patients (81 (91%) vs. 21 (75%), *p* = 0.047, *ϕ* = 0.20). Congestive heart failure (CHF)/coronary artery disease (CAD) (*p* = 0.020, *ϕ* = 0.22), bronchial asthma (*p* = 0.006, *ϕ* = 0.25), chronic obstructive pulmonary disease (COPD) (*p* = 0.018, *ϕ* = 0.24), and malignancy (0.002) were higher in the high-risk group. Oxygen saturation was lower in this group (90% (ranges: 76–98%) vs. 92.5% (ranges: 90–98%), *p* < 0.001, *r*_b_ = 0.50). The thrombolytic therapy utilization (3.6% vs. 5.6%, *p* > 0.999) and mortality rates (0% vs. 14.6%, *p* = 0.036, *ϕ* = 0.20) were higher in the high-risk group compared to the low-risk group.

A comparison of the laboratory findings for the groups is presented in Table 2. D-dimer (2083 (1165.75–4736.5) vs. 3583 (2055–7904.5), *p* = 0.046, *r*_b_ = 0.25), urea (33.5 (26.63–37.25 vs. 36 (30–49), *p* = 0.021, *r*_b_ = 0.29) and creatine kinase MB isoenzyme (CK-MB) (1.08 (0.78–1.52) vs. 1.60 (1.02–2.90), *p* = 0.017, *r*_b_ = 0.36) were high, while lymphocytes (2.31 ± 1.04 vs. 1.71 ± 0.84, *p* = 0.003, Cohen’s *d* = 0.35), hemoglobin (12.96 ± 2.11 vs. 11.72 ± 2.09, *p* = 0.008, Cohen’s *d* = 0.33), and hematocrit (38.97 ± 5.87 vs. 36.10 ± 6.01, *p* = 0.031, Cohen’s *d* = 0.48) were low. No differences were found in other laboratory data.

The patients’ radiological findings were compared between the low- and high-risk groups according to the sPESI (Table 3). Although the high-risk group exhibited a higher median CT obstruction index value compared to the low-risk group (30 (12.5–50) vs. 27.5 (14.38–53.13)), no statistically significant difference was observed between the groups (0.674).

Although AoD, PAD, LPA, IVSD, IVCR, infarct, and the PAD/AoD ratio were higher in the high-risk group compared to the low-risk group, no statistically significant difference was found between the groups. A statistically significant difference was observed only in the LPA measurement between the groups. The LPA was 18.96 ± 3.86 in the high-risk group, while it was 17.21 ± 2.64 in the low-risk group (*p* = 0.009, Cohen’s *d* = 0.53) (Table 3).

The patients’ CTPA findings, according to their six-month survival rates, are compared in Table 4. After six months of follow-up, 13 patients had died (11.11%), and 104 (88.89%) were alive. In the patients’ radiologic findings, the AoD (31.94 ± 4.80, 34.77 ± 4.49, *p* = 0.046, Cohen’s *d* = 0.59) and LPA (18.30 ± 3.49, 20.46 ± 4.61, *p* = 0.044, Cohen’s *d* = 0.60) were higher in the deceased group. There was no statistically significant difference in the mean CTOI (31.25 (12.50–50), 20 (12.5–27.5), *p* = 0.242), MPA (28.33 ± 4.72, 28.00 ± 5.42, *p* = 0.817), and PAD/AoD > 1 (70.2% vs. 92.3%, *p* = 0.110), IVSD (15.4% vs. 0%, *p* = 0.214) and IVCR (18.3% vs. 8.3%, *p* = 0.688).

A comparison of the patients’ clinical and radiologic findings with CTOI in CTPA is shown in Table 5. The CTOI was higher in those with IVSD (*p* < 0.001, *r*_b_ = 0.54) and those who received thrombolytic therapy (*p* = 0.013, *r*_b_ = 0.60). There was no difference in CTOI in relation to the aorta/MPA ratio (*p* = 0.147), IVCR (*p* = 0.438), infarcts (*p* = 0.818), mortality (*p* = 0.242), and recurrent embolism (*p* = 0.556).

## 4. Discussion

In acute pulmonary embolism (APE), validated clinical and radiological scoring systems are essential for predicting prognosis and mortality. In our study, although the CTOI index was observed to be higher in the high-risk APE patient group compared to the low-risk group, the difference between the groups did not reach statistical significance. Similarly, the RVD parameters evaluated as secondary outcomes showed insufficient clinical benefit for mortality prediction or risk stratification of high-risk populations. Importantly, we demonstrated that the sPESI outperforms radiological findings in predicting both mortality and clinical outcomes.

Risk stratification for normotensive patients diagnosed with PE should aim to distinguish low-risk patients from intermediate-risk–high-risk patients with preserved systemic arterial pressure and a high risk of a complicated course [3]. Numerous studies have confirmed the prognostic accuracy of the PESI [10,11,12]. The sPESI, which was created because the PESI scoring method is highly parametric and impractical to use, has been shown to successfully predict 30-day mortality after acute symptomatic PE. In addition, when compared with the PESI, the sPESI has similar prognostic accuracy [3]. Patients with a low-risk sPESI are unlikely to have an early adverse outcome and do not need additional imaging examinations or laboratory tests to predict their risk of adverse outcomes. Studies support the idea that, compared with imaging and laboratory biomarkers or combinations thereof, standardized clinical scores more accurately identify patients at low risk of fatal and non-fatal adverse medical outcomes in the acute phase after PE diagnosis [13,14,15,16]. It has also been reported that the sPESI score is associated with long-term mortality [17]. In our study, 89 patients were in the high-risk group according to the sPESI risk classification. The mortality rate was significantly higher in the high-risk group, as reported in the literature. Our findings suggest that clinical scoring may be more effective than laboratory and imaging methods in identifying high-risk patients and detecting possible adverse events related to them.

The factors known to be associated with an increased risk of mortality in patients with pulmonary embolism include persistent hypotension and signs of right ventricular dysfunction. Enzymes, markers of cardiac damage, and echocardiography were independent risk factors in these patients [18,19]. However, it is important to use standardized and easily accessible radiological parameters in such patients because it is not clear how a prognostic classification can be made in the case of normal blood pressure, demonstrating the signs of right ventricular dysfunction via an echocardiogram is not always possible, and there are differences in personal interpretation.

The pulmonary artery obstruction index (CTOI), when determined using pulmonary arterial computed tomography, may be important in determining the severity of an embolism, indicating the burden of thrombi, and determining treatment protocols [20]. The cardiovascular consequences of APE should be assessed not only as a consequence of the degree of pulmonary vascular obstruction but also by the degree to which it requires the right ventricle to function as a high-pressure pump. Therefore, obstruction and right ventricular reserve determine the clinical outcome [21]. Our study investigated the relationship between CTPA parameters and clinical and laboratory data, as well as the relationship between the CTOI and patient outcomes. Our results suggest that although the quantitative determination of the thrombus burden in the pulmonary artery is valuable in evaluating PE and guiding treatment strategies, its predictive value alone may be insufficient to determine the patient’s prognosis. Studies have failed to show an association between a high obstruction index and mortality and an unstable hemodynamic picture, and our ability to predict mortality is limited [21,22,23,24,25,26,27]. Akhoundi et al. also stated that the Qanadli score should be modified due to its inadequacy in predicting mortality [28]. In a study using the Mastora score, an alternative method used to assess pulmonary vascular occlusion, it was emphasized that thrombotic occlusion was not associated with the clinical severity of PE [26]. There are also studies that demonstrate that the CTOI is effective in predicting mortality. Kumamaru et al. reported that CTPA-based thrombus burden scoring is superior to the PESI in predicting 30-day mortality [29]. The CTOI has been reported to be strongly predictive for high-risk patients [30,31,32,33]. Although the results of these studies are contradictory, recent publications have shown that the CTOI score alone is insufficient in predicting mortality, similar to our study. However, when combined with clinical data, it may help physicians choose the most appropriate approach for the management of patients.

IVSD has been reported to indicate RV dysfunction [33,34,35,36,37,38]. Although one study reported that IVSD is associated with short-term mortality, defined as in-hospital death or death within 30 days [39], most studies have reported that IVSD does not predict the likelihood of mortality being caused by acute PTE [7,8,40,41]. In our study, there was no difference in mortality in the 16 (13.8%) patients with IVSD at the 6-month follow-up, as reported in most studies. In addition, there were no differences in the use of thrombolytic therapy in this group. Since IVSD is not specific to acute PE and can be found in many diseases that cause increased pulmonary artery pressure, a more careful evaluation of IVSD may be valuable. However, using it with other findings to predict clinical outcomes may be more appropriate, as in the CTOI.

Some reports have shown that the pulmonary trunk diameter may predict the severity of PE and its associated mortality [27,33,42,43]. There are also publications that suggest that high LPA is an independent risk factor for PE-related 30-day mortality [32]. In our study, there was a significant difference between the low- and high-risk groups in terms of LPA. The PAD/AoD ratio was significantly higher in the group diagnosed with APE and mortality compared to the healthy group [17]. In another study investigating the PAD/AoD ratio in acute PTE, the adequate accuracy of the PAD/AoD ratio in diagnosing moderate or severe pulmonary hypertension could not be demonstrated [44]. It was also reported that there was no correlation between the pulmonary artery diameter and echocardiographically estimated pulmonary artery pressure [34]. In many studies, the pulmonary trunk diameter was not associated with mortality, with it being found that pulmonary hypertension on CTPA alone cannot be used to diagnose severe PE [34,39,41,45,46]. We think that the PAD/AoD ratio should not be used alone; this is because there is no cut-off value for the PAD/AoD ratio and no correlation between the PAD/AoD ratio and ECHO used to measure pulmonary artery pressure. The prognostic effect of a high LPA diameter, as shown in our study, will need to be supported by further studies.

Some of the CT parameters used to detect right ventricular failure require reformatted images for measurement, require calculation, and cannot be standardized for various reasons, such as being affected by respiratory or cardiac cycles or requiring time for measurement. In contrast, venous cava inferior (VCI) contrast agent reflux (VCIR) can be evaluated quickly from the original axial sections, can be graded semiquantitatively, and does not require special reformatting. A high degree of contrast reflux may capture a higher degree of RV physiologic impairment and identify a more severe group of patients [47]. Despite identifying IVCR as a predictor of mortality in patients with PE [21,27,32,40,41,45], IVCR is unable to identify patients at risk of early death [47,48] There are also publications stating that severe PTE cannot be differentiated from non-severe PTE [34]. Again, various factors such as heart failure, tricuspid regurgitation (TR), pulmonary hypertension, and atrial fibrillation may contribute to contrast reflux. In addition, the amount of contrast given during the evaluation of reflux and the speed at which it is given may change the results of this finding. Significant interindividual variability in the reproducibility of CT findings regarding RV dysfunction has also been reported. This may partly explain the variability in published results when aiming to predict adverse outcomes based on findings related to pulmonary CTA [8,49]. In our study, the clinical course of 20 (17.2%) patients with IVCR did not differ from those without IVCR. We think that IVCR alone may be insufficient to show the course of the disease and mortality, and may be affected by many factors, as mentioned above.

One of the limitations of our study is that it was retrospective. Another was that the evaluated CTPA findings did not correlate with a reference method such as echocardiography. However, considering that previous studies have established a good correlation between echocardiographic and CT findings, we assumed that our results correlated with echocardiographic findings [7,21,34,35,36,45,50]. In addition, different generations of CT scanners were used during our study, which could be considered a limitation. Finally, although our study reports mortality rates, it does not attempt to identify the causes of these deaths. Investigating causal relationships will require a more focused and forward-looking research methodology.

## 5. Conclusions

In our study, although the CTOI values of the high-risk group in APE patients were higher than those of the low-risk group, no statistically significant difference was detected between the groups. Similarly, the findings of RVD evaluated within the scope of our secondary outcomes also did not show sufficient clinical performance in predicting mortality or identifying high-risk patients. The current data do not support the clinical use of CTOI and RVD for mortality prognosis or risk stratification in APE. However, considering the relatively limited sample size and single-center design of our study, these findings need to be validated with larger-scale, multicenter cohorts and studies involving heterogeneous populations. Particularly, with statistically powerful analyses to be conducted in larger samples, the potential prognostic value of CTOI or its synergistic effects with RVD can be re-evaluated.

## Figures and Tables

**Table 1:** Patient characteristics of the study cohort.

	sPESI Groups			
	Low Risk (*n* = 28)	High Risk (*n* = 89)	*p*-Value	**ES**
Age (years), mean ± SD	52.43 ± 12.79	66.71 ± 15.83	<0.001 ^1^	0.94
Gender (F/M), *n* (%)	14 (50)/14 (50)	53 (59.6)/36 (40.4)	0.502 ^2^	0.08
BMI (kg/m^2^), mean ± SD	29.46 ± 3.54	27.88 ± 5.12	0.073 ^3^	0.36
Saturation, median (min–max)	92.5 (90–98)	90 (76–98)	<0.001 ^4^	0.50
Comorbidity distributions, *n* (%)				
Presence of comorbidities	21 (75)	81 (91)	0.047 ^5^	0.20
Diabetes mellitus	6 (21.4)	13 (14.6)	0.391 ^5^	0.08
Hypertension	12 (42.9)	58 (65.2)	0.060 ^2^	0.19
Atrial Fibrillation	1 (3.6)	12 (13.6)	0.184 ^5^	0.14
CHF/CAD	0 (0)	15 (16.9)	0.020 ^5^	0.22
Bronchial Asthma	0 (0)	19 (21.3)	0.006 ^5^	0.25
COPD	1 (3.6)	24 (27)	0.018 ^2^	0.24
Malignancy	0 (0)	28 (31.5)	0.002 ^2^	0.31
Chronic Renal Failure	0 (0)	2 (2.2)	>0.999 ^2^	0.07
Rheumatoid Arthritis	6 (21.4)	14 (15.7)	0.566 ^5^	0.06
Symptom distribution, *n* (%)				
Dyspnea	20 (71.4)	79 (88.8)	0.037 ^5^	0.21
Syncope/Presyncope	4 (14.3)	14 (15.7)	>0.999 ^5^	0.02
Hemoptysis	3 (10.7)	13 (14.6)	0.759 ^5^	0.05
Fatigue	3 (10.7)	19 (21.3)	0.328 ^2^	0.12
Angina Pectoris	10 (35.7)	21 (23.6)	0.307 ^2^	0.12
Palpitations	12 (42.9)	28 (31.5)	0.379 ^2^	0.10
Leg Swelling	2 (7.1)	18 (20.2)	0.152 ^5^	0.15
Etiologic Factor distributions, *n* (%)				
Hormone therapy	0 (0)	15 (16.9)	0.020 ^5^	0.22
Use of oral contraceptive	0 (0)	2 (2.2)	>0.999 ^5^	0.07
Prolonged travel/immobility	11 (39.3)	18 (20.2)	0.074 ^2^	0.19
Trauma	0 (0)	2 (2.2)	>0.999 ^5^	0.07
Past surgery	8 (28.6)	27 (30.3)	>0.999 ^5^	0.02
Genetics	5 (17.9)	11 (12.4)	0.530 ^5^	0.07
Obesity	2 (7.1)	4 (4.5)	0.628 ^5^	0.05
Idiopathic	6 (21.4)	18 (20.2)	>0.999 ^5^	0.01
Deep Vein Thrombosis, n (%)	5 (17.9)	21 (24.1)	0.666 ^2^	0.06
Relapsing embolism, *n* (%)	3 (10.7)	7 (7.9)	0.701 ^5^	0.04
Thrombolytic, *n* (%)	1 (3.6)	5 (5.6)	>0.999 ^5^	0.04
Mortality	0 (0)	13 (14.6)	0.036 ^5^	0.20

^1^ Independent sample *t*-test, ^2^ Yates continuity corrected chi-square test, ^3^ Welch’s *t*-test, ^4^ Mann–Whitney U test, ^5^ Fisher’s exact test. Effect size (ES) is presented as Cohen’s *d*, Phi (*ϕ*) coefficient, and Rank biserial correlation coefficient (*r*_b_). Abbreviations: CHF/CAD: Congestive Heart Failure/Coronary Artery Disease; COPD: Chronic Obstructive Pulmonary Disease.

**Table 2:** Comparison of laboratory parameters.

	sPESI Groups			
	Low Risk (*n* = 28)	High Risk (*n* = 89)	*p*-Value	ES
Laboratory Findings				
D-dimer	2083 (1165.75–4736.5)	3583 (2055–7904.5)	0.046 ^4^	0.25
C-reactive protein	19.5 (4.5–46.75)	25 (5–67)	0.410 ^4^	0.10
Lactate Dehydrogenase	296 (209–376)	274 (225–329.5)	0.957 ^4^	0.01
Creatine kinase MB isoenzyme	1.08 (0.78–1.52)	1.60 (1.02–2.90)	0.017 ^4^	0.36
Troponin	9.43 (3.77–25.75)	26.30 (7.92–43.70)	0.054 ^4^	0.29
Lymphocytes	2.31 ± 1.04	1.71 ± 0.84	0.003 ^1^	0.35
Lactate	2.5 (1.4–3.3)	1.8 (1.5–3)	0.644 ^4^	0.07
Alanine aminotransferase	21.5 (14.75–51)	22 (12–29)	0.111 ^4^	0.20
Aspartate transferase	24 (16.75–36.25)	23 (16–34)	0.643 ^4^	0.06
Creatine	0.87 (0.68–0.91)	0.81 (0.64–1.04)	0.985 ^4^	0.01
Urea	33.5 (26.63–37.25)	36 (30–49)	0.021 ^4^	0.29
Hemoglobin	12.96 ± 2.11	11.72 ± 2.09	0.008 ^1^	0.33
Haematocrit	38.97 ± 5.87	36.10 ± 6.01	0.031 ^1^	0.48
Mean corpuscular hemoglobin concentration	33.22 ± 1.56	32.43 ± 1.34	0.012 ^1^	0.27
Red cell distribution width	13.60 (13.15–15.25)	14.90 (14–16.80)	0.002 ^4^	0.39
White blood count	9.40 (6.89–11.65)	8.81 (6.53–11.50)	0.493 ^4^	0.09
Homocysteine	13.21 ± 6.12	14.53 ± 6.05	0.374 ^1^	0.12
Protein-C	98.97 ± 33.41	102.60 ± 32.26	0.644 ^1^	0.06
Protein-S	85.15 ± 22.39	81.25 ± 25.46	0.511 ^1^	0.08
Antithrombin III	105.32 ± 12.49	99.53 ± 20.09	0.192 ^1^	0.22

^1^ Independent sample *t*-test, ^4^ Mann–Whitney U test. Effect size (ES) is presented as Cohen’s *d* and Rank biserial correlation coefficient (*r*_b_).

**Table 3:** Comparison of radiologic parameters.

	sPESI Groups			
	Low Risk (*n* = 28)	High Risk (*n* = 89)	*p*-Value	ES
**Radiological parameters**				
Computed tomography obstruction index	27.5 (14.38–53.13)	30 (12.5–50)	0.674 ^4^	0.05
Aortic diameter	31.07 ± 5.07	32.63 ± 4.73	0.138 ^1^	0.32
Pulmonary artery diameter	27.93 ± 3.87	28.40 ± 5.05	0.648 ^1^	0.10
Right pulmonary artery	18.25 ± 3.53	18.52 ± 3.79	0.742 ^1^	0.07
Left pulmonary artery	17.21 ± 2.64	18.96 ± 3.86	0.009^3^	0.53
Interventricular septal deviation	5 (17.9)	11 (12.5)	0.532 ^5^	0.07
Inferior vena cava reflux	5 (17.9)	15 (17)	>0.999 ^5^	0.01
Infarct	8 (28.6)	24 (27.3)	>0.999 ^2^	0.01
PAD/AoD (>1)	20 (71.4)	65 (73)	>0.999 ^2^	0.02

^1^ Independent sample *t*-test, ^2^ Yates continuity corrected chi-square test, ^3^ Welch’s *t*-test, ^4^ Mann–Whitney U test, ^5^ Fisher’s exact test. Effect size (ES) is presented as Cohen’s *d*, Phi (*ϕ*) coefficient, and Rank biserial correlation coefficient (*r*_b_). Abbreviations: PAD/AoD: Ratio of pulmonary artery diameter to aortic diameter.

**Table 4:** Comparison of the radiologic findings in patients with and without mortality.

	Mortality			
	Alive (*n* = 104)	Died (*n* = 13)	*p*-Value	ES
Radiological parameters				
Computed tomography obstruction index	31.25 (12.50–50)	20 (12.5–27.5)	0.242 ^4^	0.20
Aortic diameter	31.94 ± 4.80	34.77 ± 4.49	0.046 ^1^	0.59
Pulmonary artery diameter	28.33 ± 4.72	28.00 ± 5.42	0.817 ^1^	0.07
Right pulmonary artery	18.38 ± 3.71	19.08 ± 3.90	0.523 ^1^	0.19
Left pulmonary artery	18.30 ± 3.49	20.46 ± 4.61	0.044 ^1^	0.60
Interventricular septal deviation	16 (15.4)	0 (0)	0.214 ^5^	0.14
Inferior vena cava reflux	19 (18.3)	1 (8.3)	0.688 ^5^	0.08
Infarct	29 (27.9)	3 (25)	>0.999 ^5^	0.02
PAD/AoD (>1)	73 (70.2)	12 (92.3)	0.110 ^5^	0.16

^1^ Independent sample *t*-test, ^4^ Mann–Whitney U test, ^5^ Fisher’s exact test. Effect size (ES) is presented as Cohen’s *d*, Phi (*ϕ*) coefficient, and Rank biserial correlation coefficient (*r*_b_). Abbreviations: PAD/AoD: Ratio of pulmonary artery diameter to aortic diameter.

**Table 5:** The relationship between the computed tomography obstruction index and other indexes.

		*n*	Computed Tomography Obstruction Index	*p*-Value	ES
PAD/AoD	<1	91	25 (12.5–50)	0.147	0.19
	≥1	26	37.5 (25–50)		
Interventricular septal deviation	No	100	25 (11.88–50)	<0.001	0.54
	Yes	16	56.25 (45.63–75)		
Inferior vena cava reflux	No	96	27.5 (12.5–50)	0.438	0.11
	Yes	20	33.75 (16.25–55)		
Infarct	No	84	30 (12.5–50)	0.818	0.03
	Yes	32	26.25 (12.5–50)		
Thrombolytic therapy	Not given	111	27.5 (12.5–50)	0.013	0.60
	Given	6	56.25 (50–71.88)		
Mortality	No	104	31.25 (12.5–50)	0.242	0.20
	Yes	13	20 (12.5–27.5)		
Relapsing Embolism	No	107	27.5 (12.5–50)	0.556	0.11
	Yes	10	37.5 (15.63–50)		

Abbreviations: PAD/AoD: Ratio of pulmonary artery diameter to aortic diameter. Effect size (ES) is presented as Rank biserial correlation coefficient (*r*_b_).

## Data Availability

The data presented in this study are available upon request from the corresponding author.

## Abbreviations

PE	Pulmonary Embolism
CTOI	Computed Tomography Obstruction Index Ratio
sPESI	Simplified Pulmonary Embolism Severity Index
CTPA	Computed Tomography Pulmonary Angiography
AoD	Aortic Diameter
LPA	Left Pulmonary Artery
RPA	Right Pulmonary artery
VTE	Venous Thromboembolism
BNP	Brain Natriuretic Peptide
H-FABP	Heart-Type Fatty-Acid-Binding Protein
RV	Right Ventricular
TTE	Transthoracic Echocardiography
PAD	Pulmonary Artery Diameter
PAD/AoD	Pulmonary Artery to Aortic Diameter Ratio
IVSD	Interventricular Septal Deviation
IVCR	Inferior Vena Cava Reflux
V/Q	Ventilation–Perfusion Scintigraphy
MDCT	Multidetector Computed Tomography
HT	Arterial Hypertension
COPD	Chronic Obstructive Pulmonary Disease
BMI	Body Mass Index
DVT	Deep Vein Thrombosis
AF	Atrial Fibrillation
CHF/CAD	Congestive Heart Failure/Coronary Artery Disease
BA	Bronchial Asthma
CRF	Chronic Renal Failure
RA	Rheumatoid Arthritis
CK-MB	Creatine Kinase MB Isoenzyme
CRP	C-Reactive Protein
LDH	Lactate Dehydrogenase
CK-MB	Creatine Kinase MB Isoenzyme
ALT	Alanine Aminotransferase
AST	Aspartate Transferase
MCHC	Mean Corpuscular Hemoglobin Concentration
RDW	Red Cell Distribution Width
WBC	White Blood Count
AT-3	Antithrombin III
